# Pharmacological effects of different ginger juices on the concurrent symptoms in animal models of functional dyspepsia: A comparative study

**DOI:** 10.1002/fsn3.990

**Published:** 2019-06-17

**Authors:** Ling‐yun Zhong, Heng‐li Tong, Jing Zhu, Mu Lv

**Affiliations:** ^1^ School of Pharmacy Jiangxi University of Traditional Chinese Medicine Nanchang China

**Keywords:** dried ginger juice, fresh ginger boiled juice, fresh ginger juice, gastrointestinal effects, gingerol

## Abstract

**Objective:**

Patients with gastrointestinal disorders commonly suffer from poor treatment outcomes and adverse effects of traditional pharmacological therapy. Herbal medicine is a favorable alternative due to the low risk of side effects. This study was performed to explore the antiemetic effects and the improvement effect on gastrointestinal function of components of three ginger juice excipients.

**Methods:**

The compositions were analyzed by liquid chromatograph mass spectrometer (LC‐MS), especially the gingerols of dried ginger juice (DGJ), fresh ginger juice (FGJ), and fresh ginger boiled juice (FGBJ). Furthermore, the respective gastrointestinal effects on rat models with functional dyspepsia (FD) were compared.

**Results:**

The 6‐keto‐PGF_1α_ levels in the serum of the treated groups were significantly reduced (*p < *0.05), as compared with the control group. Compared with the cisplatin group, there was an apparent reduction in kaolin intake for DGJ, FGJ, and FGBJ (*p < *0.01; *p < *0.01; *p < *0.05). The intestinal propulsive rate of the rats in the treated group was significantly higher than that in the control group (*p < *0.05). Ginger juices significantly improved gastrointestinal function in rats. Eight common components were found in DGJ, FGJ, and FGBJ, among which 6‐paradol, 10‐gingerol, and 12‐shogaol led to inhibited gastric mucosal damage function effect according to the Pearson correlation analysis. Only 6‐shogaol was found to have a positive correlation with gastrointestinal function effect through Pearson correlation analysis.

**Conclusion:**

Ginger juice should be recommended for the medicinal materials used in the treatment of concurrent symptoms of FD.

## 1 INTRODUCTION

Ginger rhizome of *Zingiber officinale Roscoe*, a widely used herbal medicine, is pungent and warm according to traditional Chinese medicine (TCM) theory. The chemical composition of biological properties of ginger primarily includes nonvolatile pungent compounds, 6‐, 8‐, 10‐gingerols and 6‐, 8‐, 10‐shogaols (Ho & Chang, 2018). Ginger has been known to prevent or arrest nausea and vomiting, diastole and protect the coronary artery, inhibit the contraction of small intestine and much more (Chatturong, Kajsongkram, Tunsophon, Chanasong, & Chootip, 2018; Thamlikitkul et al., 2017; Wu et al., 2018). In TCM, ginger is regularly used as a Ministerial drug to potentiate the Monarch drug in the treatment of the main disease or to treat the concurrent symptoms.

Gingerols and shogaols are active constituents of ginger, which are difficult to purify due to its great variety and structural similarity. Thus, it is necessary to develop a rapid analysis and identification method for ginger and its subtypes. Analysis methods have been developed, including nuclear magnetic resonance (NMR), high‐performance liquid chromatography (HPLC), and ultrahigh‐performance liquid chromatography–mass spectrometry (UHPLC‐MS) (Ghasemzadeh, Jaafar, Baghdadi, & Tayebi‐Meigooni, 2018; Li et al., 2018; Park & Jung, 2016; Rai et al., 2015). However, the HPLC technology needs to be applied in combination with the standard for qualitative analysis of unknown compounds. GC‐MS has been widely applied in ginger analysis, but gingerol is hard to be detected because of its low volatility. HPLC‐MS could integrate the strong separation ability of liquid chromatography with high sensitivity and high selectivity, providing structural information. In particular, high‐resolution time‐of‐flight mass spectrometry can provide accurate molecular weight information and deduce the formula of the specific compound. Park & Jung separated and quantified 8 major gingerols and shogaols from ginger using the ultrahigh‐performance liquid‐phase chromatography–electrospray ionization–tandem mass spectrometry (HPLC‐ESI‐MS/MS) technology (Park & Jung, 2016).

Numerous TCMs can prevent gastric mucosal damage, vomiting, and gastrointestinal movement. The inhibition effects of aqueous extract from Artemisia capillaris were investigated in terms of ROS and NF‐kB of acute gastric mucosal injury induced by ethanol (Yeo, Hwang, Kim, Youn, & Lee, 2018). Strikingly, increased 6‐keto‐PGF1α was previously demonstrated to reduce the degree of gastric mucosal injury (Kou et al., 2018). The ginger extract can prevent the pica behavior of rats induced by cisplatin (Hu et al., 2016). Tangweian decoction, a Chinese herbal medicine, can significantly promote intestinal propulsion rate in diabetic mice (Tian et al., 2017).

Based on the Chinese Pharmacopoeia (2015 version), there are two subtypes of ginger, namely fresh ginger and dried ginger. Both forms can be used to obtain ginger juice. According to the National Processing Standard of Traditional Chinese Medicine—1988, ginger includes fresh ginger juice (FGJ), fresh ginger boiled juice (FGBJ), and dried ginger juice (DGJ). However, no clear distinction was identified among the ginger juices. These ginger juices are often interchangeable when preparing a TCM formulation. In the current study, we aimed to analyze the nonvolatile components in FGJ, FGBJ, and DGJ by LC‐MS. After the rats were administered with ginger juices, the ethanol‐induced gastric mucosal damage index and the levels of IL‐8, TNFα, 6‐keto‐PGF1α, cisplatin‐induced emesis, and the intestinal propulsion rate were determined to evaluate the effect on gastroenteric motivity. The chemical compositions of these three ginger juices were compared to determine which one is the most effective in the preparation of Chinese traditional medicine.

## 2 MATERIALS AND METHODS

### 2.1 Materials

A liquid chromatography coupled with a mass selective detector was employed to analyze the nonvolatile components. In addition, a high‐speed refrigerated centrifuge and free instrument were also used. All involved chemicals were of analytical grade, including anhydrous ethanol, petroleum ether, and chloroform, which were separately purchased from Xilong Chemical Co., Ltd, and Tianjin Damao Chemical Reagent Factory. Nutritional semi‐pastes were also supplied. Kaolin was prepared as previously described. Briefly, kaolin and arabic gum were mixed at a ratio of 99:1, followed by the addition of distilled water (volume) to form a thick paste. The paste was further cut into pieces resembling regular rat chow pellets. After being cut, the pellets were dried at room temperature for 48 hr (Han et al., 2014).

Fresh forms (Foshan City, Guangdong Province) and dried forms of ginger (Qianwei City, Sichuang Province) were purchased from the Tian Qi Hall medicine material crude slices limited company (Zhangshu City, Jiangxi Province, China).

The aforementioned dried ginger was prepared for use by cutting into pieces (0.3 cm × 0.3 cm × 0.3 cm), followed by being baked at 55°C for 18 hr.

Male Sprague Dawley (*SD*) rats (weighing 180 and 220 g) were purchased from Ji'nan Peng Yue Experimental Animal Breeding Co., Ltd (Certificate No. SCXK (Lu)2014‐0007). Additionally, adult male Wistar rats and Sprague Dawley rats (Shan Dong, Certificate No. SCXK20130001), weighing between 200 and 230 g, were included in the current study. All animal experiments were carried out in accordance with guidelines evaluated and approved by the Ethics Committee of Jiangxi University of Traditional Chinese Medicine.

#### 2.1.1 Preparation of herbal decoctions

##### Fresh ginger juice

Firstly, 150 g of fresh ginger was washed and squeezed as juice. After filtering the juice, the obtained residues were resuspended in the appropriate amount of water and squeezed again. Subsequently, the obtained filtrates were combined.

##### Fresh ginger boiled juice and dried ginger juice

Next, we soaked 150 g of fresh ginger and 50 g of dried ginger, respectively, in 1 L water for 1 hr. After boiling for 30 min and filtration, the obtained residues were resuspended in water and boiled for another 30 min. The filtrate was combined and concentrated to 150 ml. The dosage ratio of fresh and dried ginger is 3:1.

##### Sample preparation

Samples were prepared by dissolving 10 ml of ginger juice in 20 ml of methanol. Next, the samples underwent ultrasonic for 10 min to break the cells. The solutions were filtered, and the filtrate was further strained through a 0.22‐μm Millipore filter.

### 2.2 Methods

#### 2.2.1 LC‐MS analytical condition

The nonvolatile components in the samples were determined using LC‐MS on an Acquity UPLC BEH C_18_ (21 mm × 50 mm × 1.75 μm). The mobile phase was as follows: (A) 0.01% formic acid in water and (in B) 100% acetonitrile; gradient (in A): 0–20 min, 5%–20% B; 20–30 min, 20%–23% B; 30–50 min, 23%–40% B; 50–60 min, 40%–90% B; 60.1–70 min, 5% B. Flow rate: 0.35 ml/min; temperature: 35°C; injection volume: 4 μL.

Subsequently, ionization spray ion source (ESI) was detected by positive mode. The gas flow rate was set to 10 L/min, while the solvent temperature was 325°C. The spray pressure was 241.3 kpa, the ion source temperature was 100°C, and the capillary voltage was 4.0 kv. First‐mass range was measured m/z 100 – 1,000, and second‐mass range was measured m/z 50 – 2,000.

#### 2.2.2 Grouping and administration

##### Ethanol‐induced gastric mucosal damage

The rats were randomly divided into five groups: the normal group, the model group, the FGJ group, the FGBJ group, and the DGJ group. FGJ, FGBJ, and DGJ were administered to rats once a day for a duration of 7 days excluding the rats in the normal and model groups, which were given 0.9% physiological saline instead of FGJ, FGBJ, or DGJ. All the rats except those in the normal group were given 1 ml absolute ethanol 1 hr after the last administration of drugs. Subsequently, all animals were anesthetized. Next, the stomachs were removed and opened along the long curvature to observe the lesions macroscopically 1 hr after being injected with 6 ml of 4% paraformaldehyde. Scoring standard was as follows: Intact gastric mucosa was scored as 0; point‐shaped bleeding, point‐shaped erosion, and strip‐shaped lesion of no more than 1 mm were all scored as 1; strip‐shaped lesion with more than 1 mm was doubly scored. Total scores of every rat were considered as the lesion index. Inhibition ratio of lesion formation (%) = (A − B)/A × 100% (A, B were the lesion indices of the model group and drug‐treated group, respectively). Gastric tissues in the same location were fixed with 4% formalin, embedded with paraffin, and stained by HE. In addition, blood samples were obtained from the abdominal aorta. IL‐8, TNFα, and 6‐keto‐PGF1α in plasma were examined by radioimmunoassay.

##### Cisplatin‐induced emesis

After acclimatization to the laboratory environment for 3 days, all animals were randomly divided into five groups, namely control, cisplatin, FGJ, FGBJ, and DGJ groups. The mice in the control group were administered with peritoneal injections and gastric lavage with normal saline. The animals in the cisplatin group received peritoneal injections of cisplatin (5 mg/kg) and gastric lavage with normal saline, while animals in the thalidomide and granisetron groups received peritoneal injections of cisplatin (5 mg/kg) and gastric lavage with, respectively, FGJ (10 ml/kg), FGBJ (10 ml/kg), or DGJ (10 ml/kg).

##### Gastrointestinal propulsion

Mice were divided into 4 groups, namely the normal, FGJ, FGBJ and DGJ groups. The mice were fastened for 12 hr prior to the experiment. Fifteen minutes after the last administration of drugs, the mice were given semi‐solid nutrition in a volume of 1 ml. The animals were then sacrificed by cervical dislocation 20 min after the meal, and the whole gastrointestinal tract was removed. The entire length of the intestines was stretched out on paper, and the distance the ink had travelled from the pylorus was expressed as the percentage of the total length of small intestine between pylorus and cecum. A total of 10 animals were used for each dose or treatment, respectively.

### 2.3 Statistical analysis

All experiments were performed at least six times. The results were presented as mean value ± standard error (*SE*). Statistical analyses were performed using the SPSS 19 software with independent‐samples *t* test (ref). A value of *p < *0.05 was considered to be statistically significant. Pearson correlation analysis was applied for the relative analysis of common ingredients in all ginger juices and their pharmacodynamics indices using the SPSS 19 software.

## 3 RESULTS

### 3.1 Analysis for chemical compositions of FGJ, FGBJ, and DGJ

**Figure 1:**
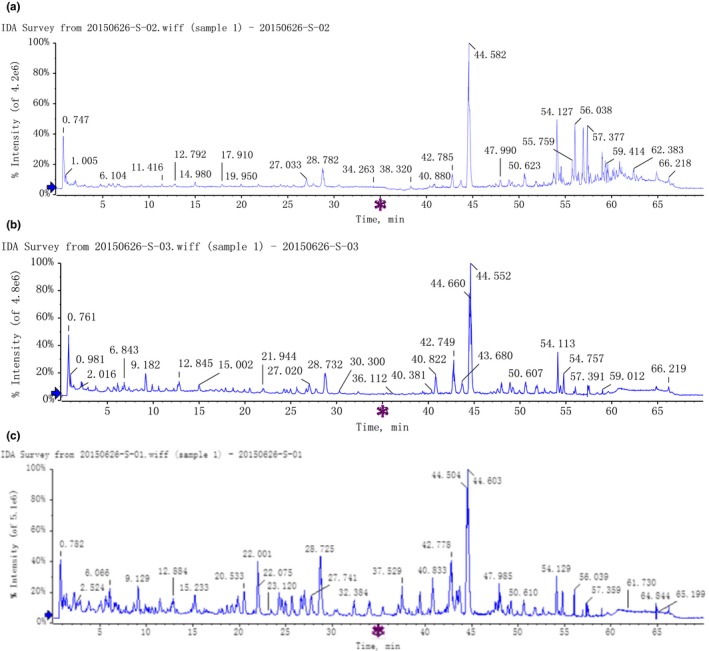
LC‐MS TIC (FGJ‐A, FGBJ‐B, DGJ‐C)

Typical total ion chromatograms (TICs) of the nonvolatile component fraction extracted from FGJ, FGBJ, and DGJ are shown in Figure 1. Although the TICs were complicated, most chromatographic peaks were noted to be well separated. Positive ion mode total ion chromatograms were analyzed according to PeakviewTM1.7. via methods of target screening and nontarget screening. The common chemical compositions of FGJ, FGBJ, and DGJ were initially identified (Table 1) (Denniff, Macleod, & Whiting, 1981; Hu, Guo, et al., 2011; Hu, Rayner, et al., 2011; Jiang, Sólyom, Timmermann, & Gang, 2010; Kikuzaki, Kawasaki, & Nakatani, 1994; Zhan, Wang, Xu, & Yin, 2008; Zhang, Gan, & Hong, 2005).

**Table 1:** Possible common chemical composition of the samples by LC‐MS

*N*	Name	TR/min	Formula	MS	Error (ppm)	Area%		
						DGJ	FGJ	FGBJ
1	4‐shogaol	27.01	C_15_H_20_O_3_	249.1484 [M + H]+	−0.5	1.75	0.89	1.83
2	6‐gingerdiol	40.84	C_17_H_28_O_4_	317.1717 [M + Na]+	−1.9	1.62	1.60	5.27
3	6‐gingerol	44.56	C_17_H_26_O_4_	295.1896 [M + H]+	−1.9	7.54	19.59	13.83
				317.1717 [M + Na]+	−2.7			
4	6‐shogaol	44.57	C_17_H_24_O_3_	277.1792 [M + H]+	−2.2	0.90	0.57	1.24
5	methyl‐6‐gingerol	50.61	C_18_H_28_O_4_	331.1875 [M + Na]+	−1.3	0.82	1.61	1.91
6	3‐or 5‐acetoxy‐6‐gingerdiol	51.81	C_19_H_30_O_5_	361.1982 [M + Na]+	−1.1	0.63	0.65	0.89
7	diacetoxy‐4‐gingerdiol	52.69	C_19_H_28_O_6_	375.1771 [M + Na]+	−1.9	0.29	0.21	0.45
8	8‐shogaol	54.13	C_19_H_28_O_3_	305.2105 [M + H]+	−2.2	1.59	4.01	0
9	8‐gingerol	54.13	C_19_H_30_O_4_	345.2033 [M + Na]+	−1	1.59	5.34	2.65
10	methyl‐3‐or 5‐acetoxy‐6‐gingerdiol	54.41	C_20_H_32_O_5_	375.2134 [M + Na]+	−2.2	0.13	0.98	0.72
11	10‐gingerdione	55.67	C_21_H_32_O_4_	371.2184 [M + Na]+	−2.5	0.08	0	0
12	6‐paradol	56.1	C_17_H_26_O_3_	301.1771 [M + H]+	−1	0	4.27	0
13	1‐dehydro‐6‐gingerdione	56.33	C_17_H_22_O_4_	313.1408 [M + Na]+	−0.9	0	0.12	0.52
14	acetoxy‐8‐gingerol	56.94	C_21_H_32_O_5_	387.2124 [M + Na]+	−4.7	0.13	3.82	0
15	10‐gingerol	56.96	C_21_H_34_O_4_	373.234 [M + Na]+	−2.5	0.13	3.82	0
16	acetoxy‐10‐gingerol	58.99	C_23_H_36_O_5_	415.2441 [M + Na]+	−3.3	0	1.50	0
17	12‐shogaol	59.12	C_23_H_36_O_3_	361.2727 [M + H]+	−3	0	0.87	0
18	10‐shogaol	59.65	C_21_H_32_O_3_	355.2228 [M + Na]+	−4.4	0.14	0.72	0
19	1‐dehydro‐10‐gingerdione	60.51	C_21_H_30_O_4_	347.2204 [M + H]+	−3.8	0	1.15	0
				369.2019 [M + Na]+	−4.6			

Note

DGJ: dried ginger juice; FGJ: fresh ginger juice; FGBJ: fresh ginger boiled juice; LC‐MS: Liquid Chromatograph‐Mass Spectrometer.

### 3.2 Ethanol‐induced gastric mucosal damage

**Figure 2:**
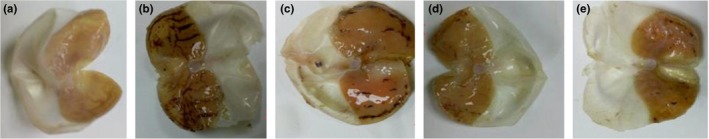
Protection of ginger juices on gastric mucosal injury induced by ethanol in rats. (a) Normal group; (b) Model group; (c) FGBJ group; (d) FGJ group; (e) DGJ group

**Figure 3:**
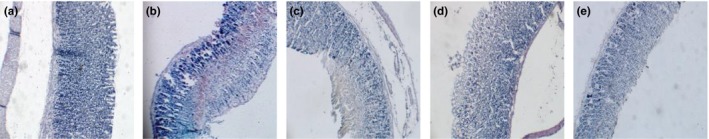
Gastric tissues. (a) Normal group; (b) Model group; (c) FGBJ group; (d) FGJ group; (e) DGJ group

Evident large bandlike hemorrhagic erosions in the glandular stomach were noted in the mode group (Figures 2 and 3, Table 2). In the FGJ, FGBJ, and DGJ groups, gastric mucosal damages were prevented in a dose‐dependent manner, when compared with the model group.

**Table 2:** Effect of ethyl acetate extract from ginger juice on gastric lesions induced by ethanol in rats

Groups	Number	UI
Normal	6	–
Model	6	64.17 ± 6.49
FGBJ	6	48.83 ± 3.31*
FGJ	6	27.33 ± 2.80*,†
DGJ	6	26.17 ± 3.60*,†

Note

DGJ: dried ginger juice; FGJ: fresh ginger juice; FGBJ: fresh ginger boiled juice; UI: ulcer index.

*p < 0.01 versus model group; ^†^ *p < *0.05 versus FGBJ group.

Compared with the control group, the gastric mucosa of rats in the model group was found to be significantly damaged. In addition, shedding, bleeding, and infiltration of inflammatory cells were apparent in the mucosa. Relative to the model group, the gastric mucosa was observed to be lighter and presented with less inflammatory cell infiltration in the ginger boiled juice group and ginger juice group, while slightly alleviated gastric mucosal lesion and inflammatory cell infiltration were noted in the ginger boiled juice group. However, the gastric was severely damaged in the dried ginger boiled juice group and ginger juice group. The aforementioned results indicate that ginger juice can inhibit the inflammation of gastric mucosa and can furthermore promote the repairing process of gastric mucosa.

The ulcer index (UI) of the groups following administration was found to be significantly lower than that in the model group (*p < *0.01). Compared with the FGBJ group, a significant reduction in the rate was revealed in the FGJ and DGJ groups (*p < *0.05). The significant reductions suggest that administration of ginger juice led to decreased intestinal propulsive rate (*p < *0.05) (Tables 2 and 3).

**Table 3:** Effect of ginger juices on IL‐8, TNFα, and 6‐keto‐PGF_1α_ activity of gastric mucosa damaged by ethanol in rats

Groups	IL‐8 (pg/ml)	TNFα (pg/ml)	6‐keto‐PGF_1α_ (pg/ml)
Normal	47.21 ± 6.73	57.70 ± 7.77	82.42 ± 7.34
Model	47.01 ± 2.34	63.90 ± 6.42	95.67 ± 7.95*
FGJ	46.27 ± 5.22	55.75 ± 8.14	81.89 ± 5.16‡
FGBJ	46.40 ± 5.05	61.35 ± 5.87	83.58 ± 10.318†
DGJ	47.11 ± 2.27	67.04 ± 6.27	84.55 ± 8.00†

Note

DGJ: dried ginger juice; FGJ: fresh ginger juice; FGBJ: fresh ginger boiled juice.

*p < 0.01 versus normal group; ^†^ *p < *0.05 versus model group; ^‡^ *p < *0.01 versus model group.

As shown in Table 3, the model group showed no significant differences relative to the control group in the content of IL‐8 (*p > *0.05). The content of TNFα was elevated, but there were no significant differences within any groups. And the content of 6‐keto‐PGF1α in serum significantly increased compared with that in the control group (*p < *0.05). This demonstrates that the acute injury stimulates the body's inflammatory response. The content of 6‐keto‐PGF1α in serum of ginger‐treated group was significantly decreased compared with the model group (*p < *0.01, *p < *0.05). Furthermore, the content of FGJ group was lower than the FGBJ group, in which it has basically returned to normal levels.

### 3.3 Cisplatin‐induced emesis

Twenty‐four hours after administering peritoneal injections of cisplatin, kaolin intake was shown to be significantly increased in the cisplatin and administered groups (*p < *0.01, vs. control group; Table 4). However, the difference in kaolin intake between the FGJ group and DGJ group reflected no statistically significant difference. After 24 hr, gradually decreasing kaolin intake was observed in the administered and cisplatin groups.

**Table 4:** Changes in kaolin intake

Groups	Kaolin	
	24 hr	48 hr
Normal	0.1731 ± 0.028	0.1556 ± 0.058
Cisplatin	3.66 ± 0.75	0.54 ± 0.18
FGJ	2.86 ± 0.68*	0.50 ± 0.14
FGBJ	1.58 ± 0.22*	0.40 ± 0.29
DGJ	2.23 ± 0.78*	0.77 ± 0.21

Note

DGJ: dried ginger juice; FGJ: fresh ginger juice; FGBJ: fresh ginger boiled juice.

* *p < *0.01 versus cisplatin group.

### 3.4 Gastrointestinal propulsion

The results showed that FGBJ, FGJ, and DGJ groups were significantly different from the normal group (*p < *0.01). Compared with the FGBJ group, the FGJ group was significantly reduced (*p < *0.05). Boiled ginger juice exhibited the strongest effect on gastrointestinal propulsion, followed by ginger boiled juice and ginger juice. Among these, FGBJ presented with the strongest effect on intestine propulsion (as shown in Table 5).

**Table 5:** Intestinal propulsive rate of groups

Group	*n*	Intestinal propulsive distance (cm)	Intestinal propulsive rat%
Normal	10	15.19 ± 1.09	28.48 ± 2.48
FGBJ	10	24.59 ± 2.18	52.01 ± 3.18*
FGJ	10	23.25 ± 1.98	48.15 ± 3.50*
DGJ	10	26.07 ± 2.54	50.66 ± 4.14*

Note

DGJ: dried ginger juice; FGJ: fresh ginger juice; FGBJ: fresh ginger boiled juice.

* *p < *0.01 versus normal group.

### 3.5 Correlation analysis of common components and efficacy index

The DGJ group with the lowest UI was indexed as 100 points. The FGJ group with the lowest concentration of TNFα in serum was taken as 100 points. The FGJ group with the lowest concentration of 6‐keto‐PGF1α in serum was taken as 100 points. The FGBJ group with the lowest kaolin intake was also indexed as 100 points. The FGBJ group with the highest intestinal propulsive rate was indexed as 100 points. In addition, the average values of the other groups were sequentially calculated. UI, TNFα, and 6‐keto‐PGF1α accounted for 10%, 45%, and 45%, respectively, for the total scores of the gastric mucosal damage function. Kaolin intake and intestinal propulsive rate accounted for 50% and 50%, respectively, for the total scores of the gastrointestinal function.

The ingredients, including 4‐shogaol, 6‐gingerol, 6‐shogaol, 8‐gingerol, 6‐paradol, 1‐dehydro‐6‐gingerdione, 10‐gingerol, and 12‐shogaol, were analyzed accordingly (Table 6). The results revealed that 6‐shogaol, 6‐paradol, 10‐gingerol, and 12‐shogaol showed significant positive correlation with gastrointestinal function and inhibited gastric mucosal damage function (*p < *0.05) (Table 7).

**Table 6:** Common ingredients (relative contents) *X_1_* to *X_8 _*and gastrointestinal comprehensive value *Y*

	*X_1_*	*X_2_*	*X_3_*	*X_4_*	*X_5_*	*X_6_*	*X_7_*	*X_8_*	*Y_1_*	*Y_2_*
DGJ	1.75%	7.54%	0.90%	1.59%	0%	0%	0%	0.13%	93.723	98.377
FGJ	0.89%	19.59%	0.57%	5.34%	0.87%	4.27%	0.12%	3.82%	99.884	95.649
FGBJ	1.83%	13.83%	1.24%	2.65%	0%	0%	0.52%	0%	94.454	100

Note

DGJ: dried ginger juice; FGJ: fresh ginger juice; FGBJ: fresh ginger boiled juice.

**Table 7:** Pearson correlation analysis

		*X_1_*	*X_2_*	*X_3_*	*X_4_*	*X_5_*	*X_6_*	*X_7_*	*X_8_*	*Y_1_*
	Pearson	−0.983	0.905	−0.801	0.986	0.994	0.994	−0.191	0.990	1
Y	Relevance	0.059	0.140	0.204	0.054	0.035	0.035	0.439	0.044	1
	Significant					*	*		*	
	*N*	3	3	3	3	3	3	3	3	

		*X_1_*	*X_2_*	*X_3_*	*X_4_*	*X_5_*	*X_6_*	*X_7_*	*X_8_*	*Y_2_*
	Pearson	0.955	−0.600	0.988	−0.793	−0.929	−0.929	0.628	−0.940	1
Y	Relevance	0.096	0.295	0.049	0.209	0.120	0.120	0.284	0.111	1
	Significant			*						
	*N*	3	3	3	3	3	3	3	3	

* Correlation is significant at the 0.05 level (1‐tailed).

## 4 DISCUSSION

The chief pungent compound of ginger is gingerol. Gingerols, unstable in heat, are prone to dehydration reaction and producing the corresponding shogaols (Jung et al., 2017), which exert antioxidant, anti‐inflammatory, antiallergic, anticancer, and antimicrobial effects (Semwal, Semwal, Combrinck, & Viljoen, 2015).

Interestingly, the gastrointestinal effect is one of its major functions. In the current study, we analyzed gingerols by LC‐MS and their gastrointestinal effects. The gingerols changed in ginger juices whatever may be their composition, and the gastrointestinal effects of ginger juices were extremely different. A total of 8 gingerols were found in DGJ, FGJ, and FGBJ, among which three, namely 6‐paradol, 10‐gingerol, and 12‐shogaol, shared a positive correlation with the inhibitory effects on gastric mucosal damage, on the basis of a Pearson correlation analysis. Notably, only 6‐shogaol presented with a positive correlation with the gastrointestinal effect. Among the four compounds, 6‐paradol and 12‐shogaol were present in FGJ only. 10‐gingerol was found in DGJ and FGJ. 6‐shogaol was found in DGJ, FGJ, and FGBJ. Importantly, the content of 6‐shogaol in each ginger juices from high to low was FGBJ, DGJ, and FGJ. The 6‐shogaol of boiled ginger juice is higher than that of unboiled ginger juice, it may be attributed to that gingerol in boiled ginger juice is transformed into shogaol after heating, and the content of gingerols in fresh ginger may be higher than that in fried ginger.

Prostaglandins are recognized as defensive repair factors of the gastric mucosa. 6‐keto‐PGF_1α_ is a stable metabolite of PG, and the content of 6‐keto‐PGF_1α_ can be used to reflect the content of PG in plasma. The current study found that all ginger juices can significantly reduce the content of 6‐keto‐PGF1α in serum. Furthermore, 6‐paradol, 10‐gingerol, and 12‐shogaol were not detected in FGBJ, which was consistent with the UI results of gastric mucosal injury induced by ethanol, revealing FGJ and DGJ have significant therapeutic effects compared with FGBJ. After 24 hr, kaolin intake in the FGBJ, DGJ, and FGJ groups was gradually decreased and showed no statistically significant difference, suggesting that all kinds of gingers can prevent vomiting effectively. In addition, all gingers can promote intestine propulsion, and FGBJ exhibits the strongest effect.

6‐Shogaol and 6‐paradol are characterized by antimicrobial activity (Galal, 2008), which may be helpful for inhibiting bacterial infection in the process of gastric mucosal injury. Additionally, 6‐shogaol and 10‐gingerol have effective antioxidant and anti‐inflammatory properties (Dugasani et al., 2010), which can prevent oxygen free radicals from acting on sulfenyl to degenerate proteins and inactivate enzymes, thus alleviating gastric mucosal damage. Moreover, the aforementioned antioxidant and anti‐inflammatory activities can ameliorate gastrointestinal injury by suppressing the inflammatory cascade reaction (Zhang, Ma, Gao, Sun, & Zhang, 2017). Currently, there are almost no specific pharmacological researches on 12‐shogaol. However, according to the result of the current study, we infer that its pharmacological effects are related to sites localized in the digestive tract and gastrointestinal injury to a certain extent. Besides, 6‐shagoal may be the most closely related substance to gastrointestinal function.

In conclusion, our findings demonstrate that when the ginger juice FGJ or DGJ could be used for the inhibition of gastric mucosal injury. Additionally, FGBJ may be the best choice for promoting gastrointestinal propulsion and all kinds of gingers are suitable for preventing vomiting.

Overall, evidence has been presented demonstrating a basic understanding of different ginger juices and their therapeutic features. Further studies should aim to explore the correlations between the components of ginger juices and other pharmacodynamic indices to reveal the entire effects of ginger juices for therapeutic regimens.

## ETHICAL STATEMENT

On behalf of, and having obtained permission from all the authors, the author declares that:

- The study's protocols and procedures were ethically reviewed and approved by Ethics Committee on Laboratory Animals of Jiangxi University of Traditional Chinese Medicine. We compliance with Laboratory Animal‐Guideline for ethical review of animal welfare and Laboratory Animal‐General requirements for animal experiment of State Standard of the People's Republic of China.
- Human testing is unnecessary in our study

## CONFLICT OF INTEREST

The authors declare that they have no conflict of interest.
